# Current use of specific wearables and factors that would motivate future use of wearables: Results based on the general German adult population

**DOI:** 10.1371/journal.pone.0349939

**Published:** 2026-06-02

**Authors:** André Hajek, Hans-Helmut König

**Affiliations:** Department of Health Economics and Health Services Research, University Medical Center Hamburg-Eppendorf, Hamburg Center for Health Economics, Hamburg, Germany; Polytechnic University of Marche: Universita Politecnica delle Marche, ITALY

## Abstract

**Background:**

A conscious use of wearables may be beneficial for health in general. Therefore, we aimed to investigate the current use of specific wearables and factors that would motivate future use of wearables in the German general adult population.

**Methodology/Principal findings:**

Data were taken from a quota-based online survey which took place in January 2026 (German general adult population, mean age of 47.3 years, 18–74 years, n = 2,591). Individuals were asked about their current use of specific wearables, and non-users were also asked about the factors that would motivate future use of wearables. Slightly more than one in three (34.8%) used one or more wearables. Based on the users, 80.8% used a smartwatch, and 24.3% used a fitness tracker, whereas only a small minority used other wearables (smart ring: 5.4%; smart clothing: 4.1%; smart glasses: 2.0%; hearables: 2.9%). Key factors that would motivate future use of wearables were (in descending order of frequency): need for medical monitoring (e.g., due to future chronic illnesses) (42.0%), lower prices (33.3%), interest in monitoring health (32.9%), health data as an incentive to lead a healthy lifestyle (27.2%), experience technological advances (18.2%), and greater user-friendliness (13.5%). Regressions showed that higher odds of currently using at least one wearable were associated with, among others, being married, living with a dog, leading a healthier lifestyle, and having more chronic conditions.

**Conclusions/Significance:**

Apart from smartwatches, there is still a lot of potential for growth in user numbers for other wearables. We recommend research in other countries and based on longitudinal data.

## Introduction

Wearables are portable devices. They are worn on the body (e.g., fitness trackers, smartwatches, or smart rings), often on the wrist [1]. Albeit still at a low level, other forms are also on the rise, such as smart glasses and smart clothing. Typical features include examining sleep quality, heart rate, or steps. More advanced devices include features such as blood pressure. Health functions are becoming increasingly valid and diverse.

In general, wearables can be beneficial for the health of users (by promoting them to stay active), e.g., in terms of higher frequency of exercises or increased duration [2]. A recent umbrella review of systematic reviews also showed that wearables can help to increase the number of steps and overall activity levels [3]. Other research showed positive effects on weight or blood pressure and even quality of life [4,5].

Thus far, the majority of studies were conducted in North America (as an overview: [1]). Only very few studies exist determining the frequency of using wearables. Based on data from late 2022, about one in four used wearables for health [6]. A German study conducted in January 2025 showed that 34.5% used at least one wearable (most often: analysis of step count/distance traveled [7]). Particularly regular sports activity and more chronic conditions were associated with higher odds of using such health functions [7].

As wearable technologies become increasingly advanced, it is relevant to examine the use of wearables with the most recent data. We also need to better understand factors that would motivate future use of wearables. In light of the very restricted knowledge, we aimed to investigate the current use of specific wearables and factors that would motivate future use of wearables in the German general adult population. We see the added value of our study somewhat in the presentation of the most up-to-date data possible, but even more so [1] in the additional examination of specific wearables that have hardly been considered thus far and [2] in the investigation of factors that could motivate the future use of wearables, which have also been little studied. Understanding the characteristics of current non-users and users of wearables is important due to the link between wearable usage and health outcomes. Moreover, a better understanding of factors that could motivate future use is important to address the potential gap in usage. A conscious use of wearables could be beneficial for the health of the population [3], which in turn could reduce pressure on the healthcare system.

## Materials and methods

### Sample

Data were used from an online survey. In total, n = 2,591 individuals participated in the survey. The only two inclusion criteria were as follows: Within the age range of 18–74 years and residing in Germany. The survey took place in January 2026. To be more precise, data collection began on January 9 and ended on January 20.

The market research company Bilendi (a large company with ISO certification) was responsible for collecting the data. Since the data comes from an online panel, we cannot rule out a certain degree of online bias (further details are presented in the limitations section).

A quota sampling method was used to ensure that the sample reflected the German general adult population aged 18–74 years in terms of age group, gender, and federal state. A comparison of our sample and the official quotas is displayed in S1 Table. This shows that our sample matches the quotas. Thus, sampling weights were not used.

This study received ethics approval from the Local Psychological Ethics Committee at the University Medical Centre Hamburg-Eppendorf (LPEK-0978). All participants gave written informed consent to participate in the study before the survey began (by agreeing to the online consent form before the survey began), which is a standard procedure for online surveys.

### Outcomes: Current use of specific wearables and factors that would motivate future use of wearables

Individuals were first asked about their current use of at least one wearable device (no or yes). Individuals replying with “yes” were asked which ones exactly (multiple answers were possible): smartwatch, fitness tracker, smart ring, smart clothing, smart glasses, or hearables. Notably, based on cognitive pre-tests, we assumed that these terms are self-explanatory in German. Therefore, we decided not to describe them in more detail.

Individuals replying with “no” were asked what would motivate them to use wearables in the future (multiple answers were possible): interest in monitoring health (e.g., steps, sleep quality), health data (e.g., number of steps) as an incentive to lead a healthy lifestyle, experience technological advances (e.g., ECG), greater user-friendliness (e.g., in terms of operation), lower prices, or need for medical monitoring (e.g., due to future chronic illnesses).

### Independent variables

In regression analysis, different sociodemographic, lifestyle, health-related, and psychosocial factors were used, based on past research (e.g., [6,7]. More precisely, previous research has shown that sociodemographic factors are associated with odds of using wearables [7,8]. This also fits in particularly well with the Unified Theory of Acceptance and Use of Technology (UTAUT) model, which, for example, considers sociodemographic factors such as age and gender to be key factors [9]. Loosely inspired by the theory of planned behavior [10], we also assume that lifestyle factors are associated with odds of using wearables. The underlying idea is that lifestyle factors may shape attitudes, social norms, and perceived control regarding the use of wearables. Moreover, health-related factors were included guided by the health belief model [11]. We assume that particularly the perceived susceptibility to health problems and the perceived severity of health risks may explain why health-related factors are associated with odds of wearable use. Behind the potential link between the psychosocial factor loneliness and odds of wearable use lies our assumption that loneliness may promote social withdrawal and a reduction in physical activity. In this respect, they may have less interest in health-related topics and thus wearables [7].

Regarding sociodemographic factors, we included age (continuous measure and additionally presented as age groups: 18–29 years, 30–39 years, 40–49, 50–59 years, 60–74 years), gender (men; women; diverse), educational level (primary education; secondary education; tertiary education; following the ISCED-97-classification [12]), labor force participation (full-time employed; retired; other), family situation (widowed; single; divorced; living separated: married or in partnership; living together: married or in partnership), urbanization level (rural; mostly urban; urban), and migration background (no; yes). Regarding lifestyle-related factors, we used frequency of sports activity (five categories from no sports activity to more than 4 hours a week), health-conscious diet (four categories from not at all to very strongly), alcohol consumption (six categories from never to daily), smoking behavior (four categories from never smoker to daily smoker), and living with a pet (not living with a pet; solely dog(s); solely cat(s); dog(s) and cat(s); other pets (but without dogs and cats)). Self-rated health (single-item, ranging from 1 = very poor to 5 = very good) and a count of 15 chronic conditions (sleep disorder; thyroid disease; diabetes; asthma; heart disease (also heart failure, cardiac insufficiency); cancer; stroke; migraine; high blood pressure; depressive disorder; dementia; joint disease (also arthrosis, rheumatism); chronic back problems; burnout; other illness) were used as health-related factors. To this end, individuals were asked to indicate any illnesses for which they had received a formal diagnosis from their doctor.

Loneliness was used as a psychosocial factor. Loneliness was quantified using the 6-item De Jong Gierveld loneliness tool [13], varying from 0 to 6, whereby higher values reflect higher levels of loneliness. Cronbach’s alpha was .79 in the present study.

### Statistical analysis

The analytic sample is first described (also stratified by current use of wearables). Then, the frequency of current use of wearables in general and specific wearables is shown (among users and by key subgroups). Afterwards, the factors associated with current use of wearables (in general) was examined by using logistic regressions. Multicollinearity was checked by using variance inflation factors (VIFs). The mean VIF was 1.80 (with the highest VIF of 3.4 for health conscious diet), suggesting that multicollinearity is not a serious concern.

We also examined the factors associated with current use of smartwatches and fitness trackers in further analysis based on logistic regressions. Subsequently, the determinants of factors motivating future use of wearables were also examined using logistic regressions. Notably, these six reasons are likely not independent of each other (correlated outcomes). However, this does not violate the assumption of the independence of observations because each of our six regressions only compares independent individuals. In future research, one could also examine the relationship between the motivators (e.g., using a multivariate probit model).

The threshold for statistical significance was set at p < 0.05. Statistical analyses were conducted using StataNow 19.5 MP-Parallel Edition (StataCorp, College Station, Texas).

## Results

### Sample characteristics

Sample characteristics are illustrated in Table 1. Mean age was 47.3 years (SD: 15.3 years; from 18 to 74 years), and 50.6% of the respondents were female. Individuals without at least one wearable differed significantly from those with at least one wearable in terms of age (group), education, marital status, employment situation, smoking behavior, alcohol intake, frequency of sports activity, health-conscious diet, living with a pet, self-rated health, and loneliness. Further details are shown in Table 1.

**Table 1:** Sample characteristics, n = 2,591 (among the total sample and stratified by wearable status).

Variables	Total sample	At least one wearable: No	At least one wearable: Yes	p-value
	N = 2591	N = 1689	N = 902	
Gender: N (%)				.14
Men	1277 (49.3%)	835 (49.4%)	442 (49.0%)	
Women	1310 (50.6%)	853 (50.5%)	457 (50.7%)	
Diverse	4 (0.2%)	1 (0.1%)	3 (0.3%)	
Age: Mean (SD)	47.3 (15.3)	47.8 (15.5)	46.4 (14.8)	.02
Age group: N (%)				<.01
18–29 years	467 (18.0%)	315 (18.7%)	152 (16.9%)	
30–39 years	463 (17.9%)	269 (15.9%)	194 (21.5%)	
40–49 years	432 (16.7%)	270 (16.0%)	162 (18.0%)	
50–59 years	559 (21.6%)	370 (21.9%)	189 (21.0%)	
60–74 years	670 (25.9%)	465 (27.5%)	205 (22.7%)	
Education: N (%)				<.01
Primary	251 (9.7%)	183 (10.8%)	68 (7.5%)	
Secondary	1161 (44.8%)	775 (45.9%)	386 (42.8%)	
Tertiary	1179 (45.5%)	731 (43.3%)	448 (49.7%)	
Marital status: N (%)				<.001
Single/Divorced/Widowed/Living separated: married/in partnership	1080 (41.7%)	788 (46.7%)	292 (32.4%)	
Living together: married/in partnership	1511 (58.3%)	901 (53.3%)	610 (67.6%)	
Employment situation: N (%)				<.001
Full-time employed	1307 (50.4%)	794 (47.0%)	513 (56.9%)	
Retired	528 (20.4%)	364 (21.6%)	164 (18.2%)	
Other	756 (29.2%)	531 (31.4%)	225 (24.9%)	
Migration background: N (%)				.44
No	2281 (88.0%)	1493 (88.4%)	788 (87.4%)	
Yes	310 (12.0%)	196 (11.6%)	114 (12.6%)	
Level of urbanization: N (%)				.07
Rural	247 (9.5%)	169 (10.0%)	78 (8.6%)	
Mostly urban	527 (20.3%)	361 (21.4%)	166 (18.4%)	
Urban	1817 (70.1%)	1159 (68.6%)	658 (72.9%)	
Smoking behavior: N (%)				<.01
Yes, daily	506 (19.5%)	346 (20.5%)	160 (17.7%)	
Yes, sometimes	285 (11.0%)	165 (9.8%)	120 (13.3%)	
No, not anymore	757 (29.2%)	479 (28.4%)	278 (30.8%)	
Never smoker	1043 (40.3%)	699 (41.4%)	344 (38.1%)	
Alcohol intake: N (%)				<.001
Daily	119 (4.6%)	73 (4.3%)	46 (5.1%)	
Several times a week	429 (16.6%)	265 (15.7%)	164 (18.2%)	
Once a week	426 (16.4%)	268 (15.9%)	158 (17.5%)	
1–3 times a month	480 (18.5%)	297 (17.6%)	183 (20.3%)	
Less often	621 (24.0%)	406 (24.0%)	215 (23.8%)	
Never	516 (19.9%)	380 (22.5%)	136 (15.1%)	
Frequency of sports activity: N (%)				<.001
No sports activity	595 (23.0%)	446 (26.4%)	149 (16.5%)	
Less than one hour a week	491 (19.0%)	343 (20.3%)	148 (16.4%)	
Regularly, 1–2 hours a week	724 (27.9%)	461 (27.3%)	263 (29.2%)	
Regularly, 2–4 hours a week	454 (17.5%)	256 (15.2%)	198 (22.0%)	
Regularly, more than 4 hours a week	327 (12.6%)	183 (10.8%)	144 (16.0%)	
Health-conscious diet: N (%)				<.001
Very strongly	265 (10.2%)	134 (7.9%)	131 (14.5%)	
Strongly	984 (38.0%)	625 (37.0%)	359 (39.8%)	
A little	1178 (45.5%)	795 (47.1%)	383 (42.5%)	
Not at all	164 (6.3%)	135 (8.0%)	29 (3.2%)	
Living with a pet: N (%)				<.001
Not living with a pet	1481 (57.2%)	1020 (60.4%)	461 (51.1%)	
Solely dog(s)	396 (15.3%)	228 (13.5%)	168 (18.6%)	
Solely cat(s)	464 (17.9%)	306 (18.1%)	158 (17.5%)	
Dog(s) and cat(s)	155 (6.0%)	76 (4.5%)	79 (8.8%)	
Other pets (but without dogs and cats)	95 (3.7%)	59 (3.5%)	36 (4.0%)	
Self-rated health (from 1 = very poor to 5 = very good): Mean (SD)	3.6 (0.8)	3.6 (0.8)	3.7 (0.8)	.02
Number of chronic conditions: Mean (SD)	1.8 (1.8)	1.8 (1.8)	1.9 (1.8)	.20
Loneliness (from 0 to 6, higher values reflect higher loneliness): Mean (SD)	3.4 (2.0)	3.5 (2.0)	3.3 (2.0)	.02

Notes: P-values are based on Chi²-tests, or independent t-tests, as appropriate.

The frequency of current use of wearables in general and specific wearables is shown in Table 2 (wearables in general) and Table 3 (specific wearables) among the total sample and by key subgroups. Overall, 34.8% of the respondents used at least one wearable (e.g., high frequencies were observed among individuals with a health-conscious diet and those living with both dog(s) and cat(s)). Among the users, 80.8% of the respondents used a smartwatch, and 24.3% used a fitness tracker (e.g., with higher frequencies among physically active individuals and those living with both dog(s) and cat(s)). In contrast, among the users, 5.4% used a smart ring, 4.1% used smart clothing, 2.0% used smart glasses, and 2.9% used hearables. Higher frequencies were particularly observed among younger individuals, individuals with a health-conscious diet, individuals living with both dog(s) and cat(s), and individuals with an unhealthy lifestyle. Additional details are shown in Table 2.

**Table 2:** Frequency of current use of wearables in general.

Variables	At least one wearable	
	No	Yes
Total sample: N (%)	1689 (65.2)	902 (34.8)
Gender: N (%)		
Male	835 (65.4)	442 (34.6)
Female	853 (65.1)	457 (34.9)
Diverse	1 (25.0)	3 (75.0)
Age group: N (%)		
18–29 years	315 (67.5)	152 (32.5)
30–39 years	269 (58.1)	194 (41.9)
40–49 years	270 (62.5)	162 (37.5)
50–59 years	370 (66.2)	189 (33.8)
60 years and older	465 (69.4)	205 (30.6)
Marital status: N (%)		
Single/Divorced/Widowed/Living together: Married/Partnership	788 (73.0)	292 (27.0)
Living separated: Married/Partnership	901 (59.6)	610 (40.4)
Education (ISCED classification): N (%)		
Primary	183 (72.9)	68 (27.1)
Secondary	775 (66.8)	386 (33.2)
Tertiary	731 (62.0)	448 (38.0)
Level of urbanization: N (%)		
Rural	169 (68.4)	78 (31.6)
Mostly urban	361 (68.5)	166 (31.5)
Urban	1159 (63.8)	658 (36.2)
Employment status: N (%)		
Full-time employed	794 (60.7)	513 (39.3)
Retired	364 (68.9)	164 (31.1)
Other	531 (70.2)	225 (29.8)
Migration background: N (%)		
No	1493 (65.5)	788 (34.5)
Yes	196 (63.2)	114 (36.8)
Smoking behavior: N (%)		
Yes, daily	346 (68.4)	160 (31.6)
Yes, occasionally	165 (57.9)	120 (42.1)
No, not anymore	479 (63.3)	278 (36.7)
No, never	699 (67.0)	344 (33.0)
Alcohol intake: N (%)		
Daily	73 (61.3)	46 (38.7)
Several times per week	265 (61.8)	164 (38.2)
Once per week	268 (62.9)	158 (37.1)
1–3 times per month	297 (61.9)	183 (38.1)
Less often	406 (65.4)	215 (34.6)
Never	380 (73.6)	136 (26.4)
Frequency of sports activity: N (%)		
Never	446 (75.0)	149 (25.0)
Less than 1 hour per week	343 (69.9)	148 (30.1)
Regularly, 1–2 hours per week	461 (63.7)	263 (36.3)
Regularly, 2–4 hours per week	256 (56.4)	198 (43.6)
Regularly, more than 4 hours per week	183 (56.0)	144 (44.0)
Health-conscious diet: N (%)		
Very strongly	134 (50.6)	131 (49.4)
Strongly	625 (63.5)	359 (36.5)
A little	795 (67.5)	383 (32.5)
Not at all	135 (82.3)	29 (17.7)
Living with a pet: N (%)		
Not living with a pet	1020 (68.9)	461 (31.1)
Solely dog(s)	228 (57.6)	168 (42.4)
Solely cat(s)	306 (65.9)	158 (34.1)
Dog(s) and cat(s)	76 (49.0)	79 (51.0)
Other pets (but without dogs and cats)	59 (62.1)	36 (37.9)

**Table 3:** Frequency of current use of specific wearables (among users).

Variables	Smartwatch		Fitness tracker		Smart ring		Smart clothing		Smart glasses		Hearables	
	No	Yes	No	Yes	No	Yes	No	Yes	No	Yes	No	Yes
Total sample: N (%)	173 (19.2)	729 (80.8)	683 (75.7)	219 (24.3)	853 (94.6)	49 (5.4)	865 (95.9)	37 (4.1)	884 (98.0)	18 (2.0)	876 (97.1)	26 (2.9)
Gender: N (%)												
Male	82 (18.6)	360 (81.4)	327 (74.0)	115 (26.0)	413 (93.4)	29 (6.6)	414 (93.7)	28 (6.3)	427 (96.6)	15 (3.4)	424 (95.9)	18 (4.1)
Female	91 (19.9)	366 (80.1)	353 (77.2)	104 (22.8)	437 (95.6)	20 (4.4)	448 (98.0)	9 (2.0)	454 (99.3)	3 (0.7)	449 (98.2)	8 (1.8)
Diverse	0 (0.0)	3 (100.0)	3 (100.0)	0 (0.0)	3 (100.0)	0 (0.0)	3 (100.0)	0 (0.0)	3 (100.0)	0 (0.0)	3 (100.0)	0 (0.0)
Age group: N (%)												
18–29 years	33 (21.7)	119 (78.3)	117 (77.0)	35 (23.0)	140 (92.1)	12 (7.9)	140 (92.1)	12 (7.9)	143 (94.1)	9 (5.9)	145 (95.4)	7 (4.6)
30–39 years	34 (17.5)	160 (82.5)	144 (74.2)	50 (25.8)	173 (89.2)	21 (10.8)	178 (91.8)	16 (8.2)	186 (95.9)	8 (4.1)	190 (97.9)	4 (2.1)
40–49 years	29 (17.9)	133 (82.1)	123 (75.9)	39 (24.1)	156 (96.3)	6 (3.7)	157 (96.9)	5 (3.1)	162 (100.0)	0 (0.0)	156 (96.3)	6 (3.7)
50–59 years	32 (16.9)	157 (83.1)	147 (77.8)	42 (22.2)	184 (97.4)	5 (2.6)	187 (98.9)	2 (1.1)	189 (100.0)	0 (0.0)	187 (98.9)	2 (1.1)
60 years and older	45 (22.0)	160 (78.0)	152 (74.1)	53 (25.9)	200 (97.6)	5 (2.4)	203 (99.0)	2 (1.0)	204 (99.5)	1 (0.5)	198 (96.6)	7 (3.4)
Marital status: N (%)												
Single/Divorced/Widowed/Living together: Married/Partnership	62 (21.2)	230 (78.8)	236 (80.8)	56 (19.2)	272 (93.2)	20 (6.8)	278 (95.2)	14 (4.8)	286 (97.9)	6 (2.1)	283 (96.9)	9 (3.1)
Living separated: Married/Partnership	111 (18.2)	499 (81.8)	447 (73.3)	163 (26.7)	581 (95.2)	29 (4.8)	587 (96.2)	23 (3.8)	598 (98.0)	12 (2.0)	593 (97.2)	17 (2.8)
Education (ISCED classification): N (%)												
Primary	12 (17.6)	56 (82.4)	54 (79.4)	14 (20.6)	63 (92.6)	5 (7.4)	64 (94.1)	4 (5.9)	66 (97.1)	2 (2.9)	67 (98.5)	1 (1.5)
Secondary	78 (20.2)	308 (79.8)	296 (76.7)	90 (23.3)	371 (96.1)	15 (3.9)	380 (98.4)	6 (1.6)	380 (98.4)	6 (1.6)	375 (97.2)	11 (2.8)
Tertiary	83 (18.5)	365 (81.5)	333 (74.3)	115 (25.7)	419 (93.5)	29 (6.5)	421 (94.0)	27 (6.0)	438 (97.8)	10 (2.2)	434 (96.9)	14 (3.1)
Level of urbanization: N (%)												
Rural	11 (14.1)	67 (85.9)	64 (82.1)	14 (17.9)	76 (97.4)	2 (2.6)	77 (98.7)	1 (1.3)	76 (97.4)	2 (2.6)	76 (97.4)	2 (2.6)
Mostly urban	30 (18.1)	136 (81.9)	130 (78.3)	36 (21.7)	160 (96.4)	6 (3.6)	164 (98.8)	2 (1.2)	163 (98.2)	3 (1.8)	161 (97.0)	5 (3.0)
Urban	132 (20.1)	526 (79.9)	489 (74.3)	169 (25.7)	617 (93.8)	41 (6.2)	624 (94.8)	34 (5.2)	645 (98.0)	13 (2.0)	639 (97.1)	19 (2.9)
Employment status: N (%)												
Full-time employed	94 (18.3)	419 (81.7)	387 (75.4)	126 (24.6)	478 (93.2)	35 (6.8)	487 (94.9)	26 (5.1)	499 (97.3)	14 (2.7)	496 (96.7)	17 (3.3)
Retired	35 (21.3)	129 (78.7)	125 (76.2)	39 (23.8)	158 (96.3)	6 (3.7)	163 (99.4)	1 (0.6)	163 (99.4)	1 (0.6)	158 (96.3)	6 (3.7)
Other	44 (19.6)	181 (80.4)	171 (76.0)	54 (24.0)	217 (96.4)	8 (3.6)	215 (95.6)	10 (4.4)	222 (98.7)	3 (1.3)	222 (98.7)	3 (1.3)
Migration background: N (%)												
No	147 (18.7)	641 (81.3)	590 (74.9)	198 (25.1)	753 (95.6)	35 (4.4)	765 (97.1)	23 (2.9)	773 (98.1)	15 (1.9)	765 (97.1)	23 (2.9)
Yes	26 (22.8)	88 (77.2)	93 (81.6)	21 (18.4)	100 (87.7)	14 (12.3)	100 (87.7)	14 (12.3)	111 (97.4)	3 (2.6)	111 (97.4)	3 (2.6)
Smoking behavior: N (%)												
Yes, daily	26 (16.2)	134 (83.8)	123 (76.9)	37 (23.1)	143 (89.4)	17 (10.6)	145 (90.6)	15 (9.4)	151 (94.4)	9 (5.6)	153 (95.6)	7 (4.4)
Yes, occasionally	34 (28.3)	86 (71.7)	80 (66.7)	40 (33.3)	105 (87.5)	15 (12.5)	101 (84.2)	19 (15.8)	113 (94.2)	7 (5.8)	116 (96.7)	4 (3.3)
No, not anymore	47 (16.9)	231 (83.1)	222 (79.9)	56 (20.1)	272 (97.8)	6 (2.2)	278 (100.0)	0 (0.0)	278 (100.0)	0 (0.0)	267 (96.0)	11 (4.0)
No, never	66 (19.2)	278 (80.8)	258 (75.0)	86 (25.0)	333 (96.8)	11 (3.2)	341 (99.1)	3 (0.9)	342 (99.4)	2 (0.6)	340 (98.8)	4 (1.2)
Alcohol intake: N (%)												
Daily	11 (23.9)	35 (76.1)	34 (73.9)	12 (26.1)	36 (78.3)	10 (21.7)	37 (80.4)	9 (19.6)	41 (89.1)	5 (10.9)	43 (93.5)	3 (6.5)
Several times per week	32 (19.5)	132 (80.5)	115 (70.1)	49 (29.9)	150 (91.5)	14 (8.5)	151 (92.1)	13 (7.9)	156 (95.1)	8 (4.9)	157 (95.7)	7 (4.3)
Once per week	35 (22.2)	123 (77.8)	115 (72.8)	43 (27.2)	150 (94.9)	8 (5.1)	148 (93.7)	10 (6.3)	157 (99.4)	1 (0.6)	157 (99.4)	1 (0.6)
1–3 times per month	34 (18.6)	149 (81.4)	139 (76.0)	44 (24.0)	176 (96.2)	7 (3.8)	181 (98.9)	2 (1.1)	181 (98.9)	2 (1.1)	181 (98.9)	2 (1.1)
Less often	34 (15.8)	181 (84.2)	172 (80.0)	43 (20.0)	210 (97.7)	5 (2.3)	214 (99.5)	1 (0.5)	213 (99.1)	2 (0.9)	207 (96.3)	8 (3.7)
Never	27 (19.9)	109 (80.1)	108 (79.4)	28 (20.6)	131 (96.3)	5 (3.7)	134 (98.5)	2 (1.5)	136 (100.0)	0 (0.0)	131 (96.3)	5 (3.7)
Frequency of sports activity: N (%)												
Never	24 (16.1)	125 (83.9)	123 (82.6)	26 (17.4)	146 (98.0)	3 (2.0)	146 (98.0)	3 (2.0)	148 (99.3)	1 (0.7)	145 (97.3)	4 (2.7)
Less than 1 hour per week	33 (22.3)	115 (77.7)	119 (80.4)	29 (19.6)	130 (87.8)	18 (12.2)	139 (93.9)	9 (6.1)	144 (97.3)	4 (2.7)	143 (96.6)	5 (3.4)
Regularly, 1–2 hours per week	48 (18.3)	215 (81.7)	204 (77.6)	59 (22.4)	250 (95.1)	13 (4.9)	253 (96.2)	10 (3.8)	258 (98.1)	5 (1.9)	256 (97.3)	7 (2.7)
Regularly, 2–4 hours per week	38 (19.2)	160 (80.8)	136 (68.7)	62 (31.3)	186 (93.9)	12 (6.1)	187 (94.4)	11 (5.6)	194 (98.0)	4 (2.0)	193 (97.5)	5 (2.5)
Regularly, more than 4 hours per week	30 (20.8)	114 (79.2)	101 (70.1)	43 (29.9)	141 (97.9)	3 (2.1)	140 (97.2)	4 (2.8)	140 (97.2)	4 (2.8)	139 (96.5)	5 (3.5)
Health-conscious diet: N (%)												
Very strongly	27 (20.6)	104 (79.4)	91 (69.5)	40 (30.5)	117 (89.3)	14 (10.7)	119 (90.8)	12 (9.2)	122 (93.1)	9 (6.9)	126 (96.2)	5 (3.8)
Strongly	79 (22.0)	280 (78.0)	259 (72.1)	100 (27.9)	336 (93.6)	23 (6.4)	340 (94.7)	19 (5.3)	353 (98.3)	6 (1.7)	351 (97.8)	8 (2.2)
A little	62 (16.2)	321 (83.8)	310 (80.9)	73 (19.1)	372 (97.1)	11 (2.9)	377 (98.4)	6 (1.6)	381 (99.5)	2 (0.5)	370 (96.6)	13 (3.4)
Not at all	5 (17.2)	24 (82.8)	23 (79.3)	6 (20.7)	28 (96.6)	1 (3.4)	29 (100.0)	0 (0.0)	28 (96.6)	1 (3.4)	29 (100.0)	0 (0.0)
Living with a pet: N (%)												
Not living with a pet	98 (21.3)	363 (78.7)	348 (75.5)	113 (24.5)	444 (96.3)	17 (3.7)	453 (98.3)	8 (1.7)	458 (99.3)	3 (0.7)	453 (98.3)	8 (1.7)
Solely dog(s)	30 (17.9)	138 (82.1)	123 (73.2)	45 (26.8)	153 (91.1)	15 (8.9)	155 (92.3)	13 (7.7)	162 (96.4)	6 (3.6)	159 (94.6)	9 (5.4)
Solely cat(s)	28 (17.7)	130 (82.3)	127 (80.4)	31 (19.6)	152 (96.2)	6 (3.8)	153 (96.8)	5 (3.2)	156 (98.7)	2 (1.3)	154 (97.5)	4 (2.5)
Dog(s) and cat(s)	7 (8.9)	72 (91.1)	59 (74.7)	20 (25.3)	69 (87.3)	10 (12.7)	68 (86.1)	11 (13.9)	72 (91.1)	7 (8.9)	74 (93.7)	5 (6.3)
Other pets (but without dogs and cats)	10 (27.8)	26 (72.2)	26 (72.2)	10 (27.8)	35 (97.2)	1 (2.8)	36 (100.0)	0 (0.0)	36 (100.0)	0 (0.0)	36 (100.0)	0 (0.0)

**Fig 1:**
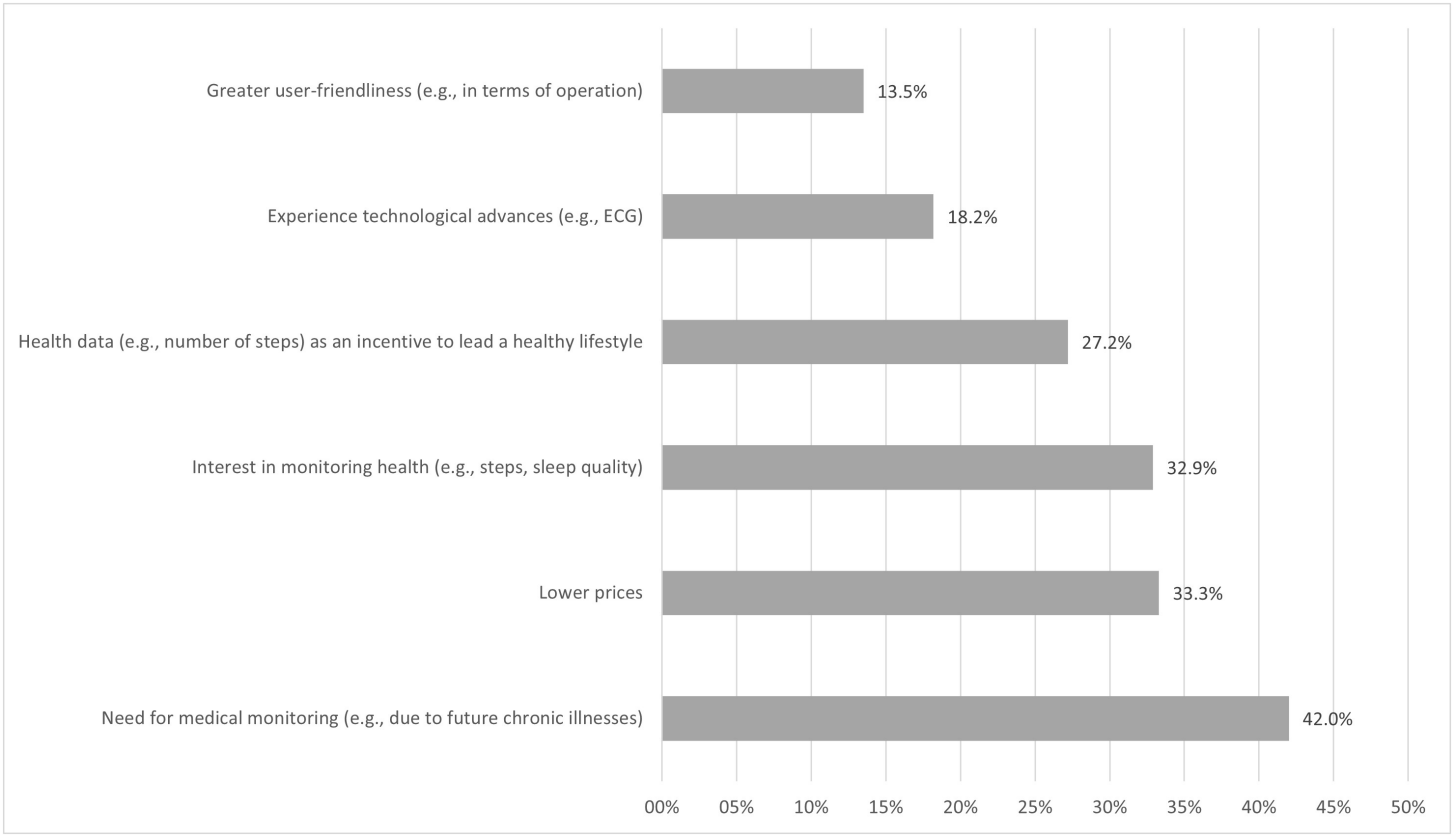
Factors motivating future use of wearables among current non-users of wearables (frequency, in %).

In descending order of frequency, factors that would motivate future use of wearables among non-users of wearables were as follows (see Fig 1): need for medical monitoring (e.g., due to future chronic illnesses) (42.0%), lower prices (33.3%), interest in monitoring health (32.9%), health data as an incentive to lead a healthy lifestyle (27.2%), experience technological advances (18.2%), and greater user-friendliness (13.5%).

### Regression analysis

The factors associated with the current use of wearables (in general) are shown in Table 4. Key fit statistics for this model are shown in S2 Table. Notably, the diverse category cannot be interpreted meaningfully due to the very few cases. Regressions showed that higher odds of currently using at least one wearable were associated with living together and being married (OR: 1.63, 95% CI: 1.36 to 1.97), being full-time employed (other vs. full-time employed, OR: 0.70, 95% CI: 0.57 to 0.87), living in an urban area (compared to rural, OR: 1.41, 95% CI: 1.04 to 1.90), living with a dog (e.g., solely dog(s) vs. not living with a pet, OR: 1.40, 95% CI: 1.10 to 1.79), higher frequency of alcohol intake (e.g., 1–3 times per month vs. never, OR: 1.49, 95% CI: 1.12 to 1.98), higher frequency of sports activity (e.g., regularly, more than 4 hours per week vs. never, OR: 1.90, 95% CI: 1.38 to 2.63), a health-conscious diet (e.g., very strongly vs. not at all, OR: 2.62, 95% CI: 1.58 to 4.37), and number of chronic conditions (OR: 1.10, 95% CI: 1.04 to 1.16). More details are shown in Table 4. Most of the determinants of current use of smartwatches and fitness trackers did not achieve statistical significance (see S3 Table).

**Table 4:** Determinants of current use of one or more wearables among the total sample. Results of multiple logistic regressions.

Independent variables	Current use of at least one wearable
Gender: Women (Ref.: Men)	1.12
	(0.93 - 1.34)
- Diverse	8.77+
	(0.76 - 101.24)
Age group: - 30–39 years (Ref.: 18–29 years)	1.30+
	(0.98 - 1.73)
40–49 years	1.13
	(0.84 - 1.53)
- 50–59 years	0.96
	(0.72 - 1.28)
- 60–74 years	0.86
	(0.61 - 1.22)
Education: - Secondary education (Ref.: Primary education)	1.23
	(0.90 - 1.69)
- Tertiary education	1.29
	(0.94 - 1.78)
Marital status: Living together: married/partnership (Ref.: Others†)	1.63***
	(1.36 - 1.97)
Employment status: - Retired (Ref.: Full-time employed)	0.85
	(0.62 - 1.17)
- Other	0.70**
	(0.57 - 0.87)
Migration background: Yes (Ref.: No)	1.15
	(0.88 - 1.50)
Level of urbanization: - Mostly urban (Ref.: Rural)	1.16
	(0.82 - 1.62)
- Urban	1.41*
	(1.04 - 1.90)
Living with a pet: - Solely dog(s) (Ref.: Not living with a pet)	1.40**
	(1.10 - 1.79)
- Solely cat(s)	1.12
	(0.88 - 1.42)
- Dog(s) and cat(s)	1.75**
	(1.22 - 2.52)
- Other pets (but without dogs and cats)	1.24
	(0.80 - 1.94)
Smoking behavior: - Yes, daily (Ref.: No, never)	0.93
	(0.72 - 1.20)
- Yes, occasionally	1.27
	(0.94 - 1.72)
- No, not anymore	1.18
	(0.95 - 1.46)
Alcohol intake: - Daily (Ref.: Never)	1.63*
	(1.04 - 2.58)
- Several times per week	1.43*
	(1.05 - 1.95)
- Once per week	1.33+
	(0.98 - 1.79)
- 1–3 times per month	1.49**
	(1.12 - 1.98)
- Less often	1.36*
	(1.04 - 1.79)
Frequency of sports activity: - Less than 1 hour per week (Ref.: Never)	1.08
	(0.81 - 1.44)
- Regularly, 1–2 hours per week	1.40*
	(1.07 - 1.81)
- Regularly, 2–4 hours per week	1.77***
	(1.32 - 2.36)
- Regularly, more than 4 hours per week	1.90***
	(1.38 - 2.63)
Health-conscious diet: - Very strongly (Ref.: Not at all)	2.62***
	(1.58 - 4.37)
- Strongly	1.74*
	(1.11 - 2.72)
- A little	1.70*
	(1.10 - 2.64)
Self-rated health (varying from 1 = very poor to 5 = very good)	0.98
	(0.86 - 1.11)
Number of chronic conditions	1.10**
	(1.04 - 1.16)
Loneliness (varying from 0 to 6; higher values indicate higher loneliness)	0.97
	(0.92 - 1.01)
Constant	0.07***
	(0.03 - 0.16)
Observations	2,591
Pseudo R²	0.06

Notes: Odds Ratios (ORs) are shown, 95% confidence intervals (CI) in parentheses; *** p < 0.001, ** p < 0.01, * p < 0.05, + p < 0.10; † Others include: single, widowed, divorced, and living separated: married/partnership.

Determinants of factors that would motivate future use of wearables among current non-users of wearables are shown in Table 5. For example, while women had higher odds of reporting interest in monitoring health compared to men, they had lower odds of reporting experience technological advances (as drivers to buy wearables). Moreover, older age groups had lower odds of reporting interest in monitoring health, reporting health data (e.g., number of steps) as an incentive to lead a healthy lifestyle, and reporting experience technological advances. In contrast, such older age groups had higher odds of reporting a need for medical monitoring (e.g., due to future chronic illnesses) as a factor to buy wearables.

**Table 5:** Determinants of factors that would motivate future use of wearables among current non-users of wearables. Results based on multiple logistic regression analysis.

Independent variables	Interest in monitoring health	Health data (e.g., number of steps) as an incentive to lead a healthy lifestyle	Experience technological advances	Greater user-friendliness	Lower prices	Need for medical monitoring
Gender: - Women (Ref.: Men)	1.31*	1.06	0.55***	0.76+	0.74**	0.86
	(1.04 - 1.64)	(0.83 - 1.35)	(0.42 - 0.73)	(0.56 - 1.04)	(0.59 - 0.93)	(0.69 - 1.08)
Age group: - 30–39 years (Ref.: 18–29 years)	0.71+	0.78	0.70+	1.36	1.13	1.30
	(0.50 - 1.01)	(0.54 - 1.12)	(0.46 - 1.06)	(0.85 - 2.17)	(0.79 - 1.63)	(0.88 - 1.91)
- 40–49 years	0.68*	0.64*	0.54**	0.53*	0.87	1.99***
	(0.48 - 0.98)	(0.44 - 0.94)	(0.35 - 0.83)	(0.30 - 0.94)	(0.60 - 1.26)	(1.36 - 2.91)
- 50–59 years	0.73+	0.71+	0.60*	0.98	0.92	2.92***
	(0.52 - 1.02)	(0.50 - 1.02)	(0.40 - 0.91)	(0.61 - 1.59)	(0.65 - 1.31)	(2.04 - 4.18)
- 60–74 years	0.57**	0.57**	0.38***	0.97	0.93	3.25***
	(0.38 - 0.85)	(0.37 - 0.87)	(0.23 - 0.64)	(0.55 - 1.69)	(0.62 - 1.39)	(2.16 - 4.89)
Education: - Secondary education (Ref.: Primary education)	1.02	0.87	1.29	0.69+	1.06	1.74**
	(0.71 - 1.46)	(0.60 - 1.28)	(0.80 - 2.08)	(0.44 - 1.07)	(0.75 - 1.50)	(1.20 - 2.52)
- Tertiary education	1.12	1.10	1.71*	0.54**	0.77	1.75**
	(0.78 - 1.61)	(0.75 - 1.62)	(1.06 - 2.75)	(0.34 - 0.85)	(0.54 - 1.10)	(1.19 - 2.55)
Marital status: Living together: married/partnership (Ref.: Others†)	1.11	1.04	1.09	1.12	0.73**	1.02
	(0.89 - 1.39)	(0.82 - 1.31)	(0.83 - 1.44)	(0.82 - 1.52)	(0.59 - 0.92)	(0.82 - 1.27)
Employment status: - Retired (Ref.: Full-time employed)	0.97	0.76	1.09	0.84	1.37+	1.03
	(0.67 - 1.41)	(0.51 - 1.15)	(0.67 - 1.78)	(0.51 - 1.40)	(0.94 - 1.97)	(0.72 - 1.46)
- Other	0.99	0.84	0.92	0.96	1.47**	1.23
	(0.77 - 1.28)	(0.64 - 1.10)	(0.67 - 1.27)	(0.67 - 1.37)	(1.13 - 1.90)	(0.95 - 1.59)
Migration background: Yes (Ref.: No)	1.17	0.89	1.33	1.40	1.27	0.78
	(0.84 - 1.62)	(0.63 - 1.27)	(0.91 - 1.94)	(0.92 - 2.13)	(0.91 - 1.76)	(0.55 - 1.10)
Level of urbanization: - Mostly urban (Ref.: Rural)	0.99	1.10	0.96	0.85	1.00	1.18
	(0.67 - 1.47)	(0.72 - 1.69)	(0.57 - 1.64)	(0.50 - 1.42)	(0.67 - 1.48)	(0.79 - 1.76)
- Urban	0.88	1.01	1.29	0.75	0.90	1.15
	(0.62 - 1.26)	(0.69 - 1.47)	(0.81 - 2.05)	(0.47 - 1.18)	(0.63 - 1.29)	(0.80 - 1.64)
Living with a pet: - Solely dog(s) (Ref.: Not living with a pet)	0.91	1.10	1.27	1.01	1.22	1.00
	(0.66 - 1.25)	(0.79 - 1.54)	(0.86 - 1.87)	(0.65 - 1.56)	(0.88 - 1.68)	(0.73 - 1.38)
- Solely cat(s)	0.83	0.73*	1.16	0.90	0.97	1.03
	(0.63 - 1.11)	(0.53 - 0.99)	(0.82 - 1.64)	(0.60 - 1.34)	(0.73 - 1.28)	(0.78 - 1.37)
- Dog(s) and cat(s)	1.17	1.08	1.34	1.65	0.91	0.90
	(0.70 - 1.93)	(0.64 - 1.84)	(0.72 - 2.47)	(0.89 - 3.09)	(0.53 - 1.57)	(0.53 - 1.53)
- Other pets (but without dogs and cats)	1.01	0.60	0.45	1.24	0.89	2.35**
	(0.57 - 1.77)	(0.30 - 1.19)	(0.17 - 1.17)	(0.58 - 2.66)	(0.49 - 1.60)	(1.32 - 4.18)
Smoking behavior: - Yes, daily (Ref.: No, never)	0.80	1.14	1.14	0.94	1.10	0.85
	(0.59 - 1.08)	(0.83 - 1.56)	(0.78 - 1.67)	(0.62 - 1.43)	(0.82 - 1.46)	(0.64 - 1.13)
- Yes, occasionally	0.77	1.08	1.93**	1.17	0.70+	0.42***
	(0.52 - 1.14)	(0.72 - 1.61)	(1.25 - 2.96)	(0.70 - 1.95)	(0.47 - 1.06)	(0.27 - 0.65)
- No, not anymore	0.89	0.99	1.14	1.14	1.01	1.02
	(0.68 - 1.16)	(0.74 - 1.32)	(0.81 - 1.60)	(0.79 - 1.65)	(0.77 - 1.33)	(0.79 - 1.33)
Alcohol intake: - Daily (Ref.: Never)	1.82*	1.80*	0.51	1.10	1.05	0.41**
	(1.05 - 3.14)	(1.02 - 3.19)	(0.23 - 1.17)	(0.52 - 2.34)	(0.61 - 1.80)	(0.23 - 0.73)
- Several times per week	1.27	1.20	1.11	0.83	0.75	0.70+
	(0.88 - 1.83)	(0.81 - 1.77)	(0.71 - 1.74)	(0.49 - 1.43)	(0.52 - 1.09)	(0.49 - 1.01)
- Once per week	0.96	0.91	1.06	1.30	0.90	0.75
	(0.67 - 1.38)	(0.62 - 1.34)	(0.68 - 1.64)	(0.80 - 2.12)	(0.63 - 1.28)	(0.52 - 1.06)
- 1–3 times per month	1.45*	1.13	0.80	1.27	0.76	0.96
	(1.04 - 2.03)	(0.79 - 1.62)	(0.52 - 1.24)	(0.79 - 2.02)	(0.54 - 1.07)	(0.69 - 1.33)
- Less often	1.01	0.98	0.94	1.14	0.86	1.08
	(0.74 - 1.37)	(0.70 - 1.37)	(0.64 - 1.39)	(0.74 - 1.75)	(0.64 - 1.17)	(0.80 - 1.46)
Frequency of sports activity: - Less than 1 hour per week (Ref.: Never)	1.46*	1.88***	1.43+	0.88	1.10	0.72*
	(1.06 - 2.00)	(1.32 - 2.67)	(0.93 - 2.18)	(0.56 - 1.38)	(0.80 - 1.50)	(0.53 - 0.99)
- Regularly, 1–2 hours per week	1.27	1.73**	1.53*	1.04	0.76+	0.76+
	(0.93 - 1.73)	(1.23 - 2.43)	(1.02 - 2.28)	(0.69 - 1.57)	(0.56 - 1.03)	(0.56 - 1.02)
- Regularly, 2–4 hours per week	1.27	1.82**	1.52+	0.69	1.09	1.03
	(0.88 - 1.82)	(1.23 - 2.68)	(0.96 - 2.41)	(0.40 - 1.16)	(0.76 - 1.55)	(0.72 - 1.46)
- Regularly, more than 4 hours per week	1.17	1.42	1.23	0.95	0.74	1.09
	(0.77 - 1.76)	(0.91 - 2.21)	(0.73 - 2.07)	(0.54 - 1.65)	(0.49 - 1.12)	(0.73 - 1.62)
Health-conscious diet: - Very strongly (Ref.: Not at all)	0.97	1.38	1.24	1.15	0.63	1.36
	(0.55 - 1.72)	(0.71 - 2.66)	(0.58 - 2.64)	(0.49 - 2.69)	(0.36 - 1.11)	(0.78 - 2.36)
- Strongly	1.07	1.70+	1.57	1.67	0.74	1.02
	(0.69 - 1.68)	(0.99 - 2.90)	(0.85 - 2.89)	(0.87 - 3.22)	(0.48 - 1.12)	(0.67 - 1.55)
- A little	1.16	1.80*	1.33	1.46	0.99	1.16
	(0.76 - 1.79)	(1.07 - 3.03)	(0.74 - 2.42)	(0.77 - 2.75)	(0.67 - 1.46)	(0.78 - 1.73)
Self-rated health (varying from 1 = very poor to 5 = very good)	1.04	1.08	1.16	1.40**	1.01	0.98
	(0.89 - 1.22)	(0.91 - 1.27)	(0.96 - 1.41)	(1.13 - 1.75)	(0.86 - 1.18)	(0.84 - 1.14)
Number of chronic conditions	1.07+	1.04	1.14**	1.18***	0.99	1.12**
	(1.00 - 1.15)	(0.97 - 1.13)	(1.04 - 1.24)	(1.07 - 1.29)	(0.92 - 1.06)	(1.04 - 1.20)
Loneliness (varying from 0 to 6; higher values indicate higher loneliness)	0.97	1.02	1.10**	1.08*	1.03	0.95+
	(0.92 - 1.03)	(0.96 - 1.08)	(1.02 - 1.18)	(1.00 - 1.17)	(0.97 - 1.09)	(0.90 - 1.00)
Constant	0.36*	0.14***	0.04***	0.03***	0.86	0.25**
	(0.14 - 0.92)	(0.05 - 0.39)	(0.01 - 0.14)	(0.01 - 0.13)	(0.34 - 2.16)	(0.10 - 0.64)
Observations	1,688	1,688	1,688	1,688	1,688	1,688
Pseudo R²	0.02	0.04	0.08	0.05	0.04	0.09

Notes: Odds Ratios (ORs) are shown, 95% confidence intervals (CI) in parentheses; *** p < 0.001, ** p < 0.01, * p < 0.05, + p < 0.10; † Others include: single, widowed, divorced, and living separated: married/partnership.

## Discussion

The aim of this study was to investigate the present use of specific wearables and factors that motivate future use of wearables in the German general adult population. Roughly one in three used at least one wearable. About four in five used a smartwatch and nearly one in four used a fitness tracker, whereas other wearables were rarely used. Different factors, such as need for medical monitoring, price issues, or interest in monitoring health, could motivate non-users to use them in the future. Several sociodemographic, lifestyle-related, and health-related factors were associated with the use of wearables. In contrast, most of the determinants of current use of smartwatches and fitness trackers did not achieve statistical significance. While the need for medical monitoring was a reason to buy wearables for older current non-users, other reasons were more important for younger non-users (e.g., experience technological advances). Our study expands our present knowledge base on the use of wearables (both general and specific wearables). Our study also identified factors that would motivate future use of wearables. In light of the restricted knowledge, we consider particularly the investigation of specific wearables (such as smart clothing or smart glasses) and the factors that could motivate the future use of wearables to be a key contribution of our work.

Our data on the frequency of use of at least one wearable (34.8%) corresponds very closely with a German study (34.5%) based on data from January 2025 [7]. Moreover, the frequencies for smartwatches [14] and fitness trackers [15] are well in line with other research from Germany. Other wearables are still more of a marginal phenomenon in Europe [16]. Previous research showed that while the user base of wearables steadily increases in Germany, it is more cautious and somewhat lower compared to the United States and certain other European countries [17,18]. It remains to be seen to what extent the less common wearables will be used in the future. The identified factors that could motivate non-users of wearables to use them in the future are supported by previous research in this area [19–21].

In line with previous research, our study showed that some sociodemographic factors (e.g., marital status or employment status) were associated with the odds of using wearables [7,8], stressing the relevance of family-related and occupational contacts for wearables.

As shown by previous research [22], living with a dog is commonly associated with more physical activity (e.g., walking). We argue that those individuals also want to measure and track these activities (e.g., step counting in wearables). Therefore, these results are very plausible from our point of view. Moreover, individuals who regularly engage in many hours of sports per week and individuals who eat a health-conscious diet had higher odds of using wearables in our study. We assume that individuals want to measure and monitor their sports activities, possibly share them (on social media platforms), and use such wearables to improve their athletic performance in a targeted manner [23–25]. As more and more apps nowadays offer ways to track the diet in minute detail, this could also be investigated in the future. Notably, higher alcohol intake (compared to never) was associated with greater odds of using wearables in our study. This may seem counterintuitive given the fact that higher alcohol consumption in itself is commonly a sign of a less healthy lifestyle, which, in turn, is related to the use of wearables [7]. However, it is important to note that completely abstaining from alcohol could also indicate that one is a recovering alcoholic or that there are other health reasons for avoiding alcohol. We would therefore like to interpret these results with due caution and encourage further research.

Individuals with chronic illnesses could find the health functions (such as monitoring blood pressure, heart rate, and sleep apnea) of wearables particularly useful for managing their conditions as effectively as possible and recognizing early warning symptoms. This may explain why chronic conditions were associated with higher odds of using wearables in our study. Such findings are also in accordance with previous research [26,27].

Among non-users, women had higher odds of reporting interest in monitoring their health (as a reason for future use of wearables) compared to men, whereas they had lower odds of reporting experiencing technological advances as a reason for future use of wearables. Such findings may reflect that women tend to be more interested in health issues, while men are more interested in technology [28–30]. As expected, older age groups were more interested in the need for medical monitoring due to future chronic illnesses. The age-related proximity to chronic diseases may play a significant role here (e.g., older adults often report higher fear of diseases such as dementia [31]). In contrast, younger individuals tend to be more interested in technological progress [32].

Our work can also be reinterpreted in terms of existing theories such as the UTAUT model, the theory of planned behavior, or the health belief model. For example, future research could also examine in detail how lifestyle factors may shape social norms or shape attitudes, which in turn could affect the odds of using wearables. Similarly, upcoming research could investigate whether health-related factors (e.g., number of chronic conditions) could affect the perceived severity of health risks, which in turn may shape the odds of using wearables.

We would like to note some strengths and limitations of this work. Data were taken from a large sample of the German general adult population (representing the demographic composition of the German population in terms of age, gender, and federal state). The current use of wearables was quantified in detail. Notably, there may be other reasons against the use of wearables (such as concerns about data protection or discomfort of sharing personal health metrics). This should be examined in future studies. Moreover, reasons for using specific wearables – which could vary significantly depending on the wearable device (e.g., smart rings vs. smart clothing) – were not recorded. Various independent variables were included. It should be emphasized that the data were taken from an online sample. Thus, the possibility of an online bias cannot be completely dismissed. Due to their affinity for online activities, the included respondents may also generally have a greater affinity for technology such as wearables. Moreover, our study had a cross-sectional design. Thus, it has shortcomings when it comes to clarifying the directionality. For example, the use of wearables could also influence social psychosocial factors such as loneliness over time. Additionally, the answers are based on self-reported information. This means that certain biases (such as recall bias) may exist. Furthermore, the possibility of a non-response bias cannot be ruled out. For example, those from the online panel who participated in the survey may have had more interest in technology than non-participants. Additionally, the low Pseudo R²-values indicate that other factors may also be relevant, which could be taken into account in future research (e.g., enthusiasm for technology).

A conscious use of wearables may be beneficial for health. Therefore, it is important to understand the current use of specific wearables and the factors that would motivate future use. Apart from smartwatches, there is still a lot of potential for growth in user numbers for other wearables. We recommend research in other countries and based on longitudinal data.

## Supporting information

S1 Table Comparison: Sample and official quotas. (DOCX)

S2 Table Measures of fit for our main model. (DOCX)

S3 Table Determinants of current use of smartwatches and fitness trackers among users of wearables. Results based on multiple logistic regression analysis. Odds Ratios are shown, 95% CI in parentheses; *** p < 0.001, ** p < 0.01, * p < 0.05, + p < 0.10; † Others include: single, widowed, divorced, and living separated: married/partnership.
(DOCX)
