# Supplementary material for: Current use of specific wearables and factors that would motivate future use of wearables: Results based on the general German adult population
Source: PLoS One. 2026 Jun 2;21(6):e0349939. doi: 10.1371/journal.pone.0349939 (PMC13229337; doi:10.1371/journal.pone.0349939)
Supplement: S1 Table — (DOCX) [file pone.0349939.s001.docx]

S1 Table. **Comparison: Sample and official quotas**

|  | Sample | Official Quotas |
| --- | --- | --- |
| Age |  |  |
| 18 - 29 years | 18% | 18% |
| 30 - 39 years | 18% | 18% |
| 40 - 49 years | 17% | 17% |
| 50 - 59 years | 22% | 22% |
| 60 - 74 years | 26% | 25% |
| Federal State |  |  |
| Baden-Württemberg | 13% | 13% |
| Bavaria | 16% | 16% |
| Berlin | 4% | 4% |
| Brandenburg | 3% | 3% |
| Bremen | 1% | 1% |
| Hamburg | 2% | 2% |
| Hesse | 8% | 8% |
| Mecklenburg-Western Pomerania | 2% | 2% |
| Lower Saxony | 10% | 10% |
| North Rhine-Westphalia | 22% | 22% |
| Rhineland-Palatinate | 5% | 5% |
| Saarland | 1% | 1% |
| Saxony | 5% | 5% |
| Saxony-Anhalt | 3% | 3% |
| Schleswig-Holstein | 3% | 3% |
| Thuringia | 3% | 3% |
| Gender |  |  |
| Female | 51% | 50% |
| Male | 49% | 50% |
