# Supplementary material for: Current use of specific wearables and factors that would motivate future use of wearables: Results based on the general German adult population
Source: PLoS One. 2026 Jun 2;21(6):e0349939. doi: 10.1371/journal.pone.0349939 (PMC13229337; doi:10.1371/journal.pone.0349939)
Supplement: S2 Table — (DOCX) [file pone.0349939.s002.docx]

S2 Table. **Measures of fit for our main model**

Log-Lik Intercept Only: -1674.512 Log-Lik Full Model: -1568.818

D(2542): 3137.635 LR(36): 211.389

Prob > LR: 0.000

McFadden's R2: 0.063 McFadden's Adj R2: 0.034

Maximum Likelihood R2: 0.078 Cragg & Uhler's R2: 0.108

McKelvey and Zavoina's R2: 0.112 Efron's R2: 0.081

Variance of y*: 3.705 Variance of error: 3.290

Count R2: 0.678 Adj Count R2: 0.074

AIC: 1.249 AIC*n: 3235.635

BIC: -16841.974 BIC': 71.564
