# Supplementary material for: Current use of specific wearables and factors that would motivate future use of wearables: Results based on the general German adult population
Source: PLoS One. 2026 Jun 2;21(6):e0349939. doi: 10.1371/journal.pone.0349939 (PMC13229337; doi:10.1371/journal.pone.0349939)
Supplement: S3 Table — Results based on multiple logistic regression analysis. Odds Ratios are shown, 95% CI in parentheses; *** p < 0.001, ** p < 0.01, * p < 0.05, + p < 0.10; † Others include: single, widowed, divorced, and living separated: married/partnership. (DOCX) [file pone.0349939.s003.docx]

S3 Table. **Determinants of current use of smartwatches and fitness trackers among users of wearables. Results based on multiple logistic regression analysis.**

| Independent variables | Current use of smartwatch | Current use of fitness trackers |
| --- | --- | --- |
|  |  |  |
| Gender: - Women (Ref.: Men) | 0.88 | 0.92 |
|  | (0.61 - 1.28) | (0.65 - 1.30) |
| Age group: - 30 to 39 years (Ref.: 18 to 29 years) | 1.18 | 1.12 |
|  | (0.67 - 2.08) | (0.66 - 1.91) |
| - 40 to 49 years | 1.09 | 1.09 |
|  | (0.60 - 2.00) | (0.62 - 1.93) |
| - 50 - 59 years | 1.23 | 0.98 |
|  | (0.67 - 2.25) | (0.56 - 1.74) |
| - 60 to 74 years | 0.95 | 1.38 |
|  | (0.46 - 1.94) | (0.70 - 2.72) |
| Education: - Secondary education (Ref.: Primary education) | 0.72 | 1.21 |
|  | (0.35 - 1.47) | (0.62 - 2.37) |
| - Tertiary education | 0.91 | 1.10 |
|  | (0.45 - 1.87) | (0.56 - 2.15) |
| Marital status: Living together: married/partnership (Ref.: Others†) | 1.14 | 1.61* |
|  | (0.77 - 1.67) | (1.10 - 2.35) |
| Employment status: - Retired (Ref.: Full-time employed) | 0.94 | 0.90 |
|  | (0.49 - 1.80) | (0.48 - 1.66) |
| - Other | 0.97 | 1.15 |
|  | (0.63 - 1.51) | (0.76 - 1.73) |
| Migration background: Yes (Ref.: No) | 0.87 | 0.64 |
|  | (0.52 - 1.46) | (0.37 - 1.09) |
| Level or urbanization: - Mostly urban (Ref.: Rural) | 0.78 | 1.33 |
|  | (0.36 - 1.68) | (0.65 - 2.70) |
| - Urban | 0.74 | 1.51 |
|  | (0.37 - 1.47) | (0.80 - 2.84) |
| Living with a pet: - Solely dog(s) (Ref.: Not living with a pet) | 1.43 | 0.99 |
|  | (0.87 - 2.35) | (0.64 - 1.54) |
| - Solely cat(s) | 1.30 | 0.71 |
|  | (0.79 - 2.12) | (0.44 - 1.14) |
| - Dog(s) and cat(s) | 3.36** | 0.84 |
|  | (1.40 - 8.02) | (0.45 - 1.56) |
| - Other pets (but without dogs and cats) | 0.69 | 1.16 |
|  | (0.31 - 1.52) | (0.53 - 2.56) |
| Smoking behavior: - Yes, daily (Ref.: No, never) | 1.10 | 0.89 |
|  | (0.63 - 1.94) | (0.53 - 1.47) |
| - Yes, occasionally | 0.56* | 1.36 |
|  | (0.32 - 0.96) | (0.81 - 2.29) |
| - No, not anymore | 1.16 | 0.73 |
|  | (0.75 - 1.80) | (0.48 - 1.09) |
| Alcohol intake: - Daily (Ref.: Never) | 0.64 | 1.17 |
|  | (0.27 - 1.54) | (0.50 - 2.76) |
| - Several times per week | 0.92 | 1.39 |
|  | (0.49 - 1.73) | (0.76 - 2.52) |
| - Once per week | 0.87 | 1.31 |
|  | (0.47 - 1.60) | (0.73 - 2.36) |
| - 1-3 times per month | 1.00 | 1.14 |
|  | (0.55 - 1.80) | (0.64 - 2.01) |
| - Less often | 1.28 | 0.93 |
|  | (0.72 - 2.29) | (0.53 - 1.63) |
| Frequency of sports activity: - Less than 1 hour per week (Ref.: Never) | 0.71 | 1.13 |
|  | (0.38 - 1.31) | (0.60 - 2.10) |
| - Regularly, 1-2 hours per week | 0.94 | 1.25 |
|  | (0.53 - 1.69) | (0.71 - 2.19) |
| - Regularly, 2-4 hours per week | 0.87 | 1.89* |
|  | (0.47 - 1.60) | (1.07 - 3.34) |
| - Regularly, more than 4 hours per week | 0.78 | 1.69 |
|  | (0.40 - 1.53) | (0.90 - 3.18) |
| Health-conscious diet: - Very strongly (Ref.: Not at all) | 0.66 | 1.22 |
|  | (0.21 - 2.08) | (0.42 - 3.56) |
| - Strongly | 0.74 | 0.98 |
|  | (0.25 - 2.16) | (0.36 - 2.69) |
| - A little | 1.08 | 0.67 |
|  | (0.37 - 3.10) | (0.25 - 1.80) |
| Self-rated health (varying from 1 = very poor to 5 = very good) | 1.06 | 1.03 |
|  | (0.83 - 1.37) | (0.81 - 1.31) |
| Number of chronic conditions | 1.00 | 1.06 |
|  | (0.89 - 1.13) | (0.95 - 1.18) |
| Loneliness (varying from 0 to 6; higher values indicate higher loneliness) | 0.98 | 1.08+ |
|  | (0.90 - 1.08) | (0.99 - 1.17) |
| Constant | 6.28* | 0.07** |
|  | (1.09 - 36.31) | (0.01 - 0.38) |
|  |  |  |
| Observations | 899 | 899 |
| Pseudo R² | 0.04 | 0.05 |

Odds Ratios are shown, 95% CI in parentheses; *** p<0.001, ** p<0.01, * p<0.05, + p<0.10; † Others include: single, widowed, divorced, and living separated: married/partnership.
